# Socioeconomic Inequalities and Toothbrushing Frequency among Schoolchildren Aged 6 to 12 Years in a Multi-Site Study of Mexican Cities: A Cross-Sectional Study

**DOI:** 10.3390/children9071069

**Published:** 2022-07-18

**Authors:** Gladys Remigia Acuña-González, Juan Alejandro Casanova-Sarmiento, Horacio Islas-Granillo, Sonia Márquez-Rodríguez, David Benítez-Valladares, Martha Mendoza-Rodríguez, Rubén de la Rosa-Santillana, José de Jesús Navarrete-Hernández, Carlo Eduardo Medina-Solís, Gerardo Maupomé

**Affiliations:** 1 School of Dentistry, Autonomous University of Campeche, Campeche 24039, Mexico; glracuna@uacam.mx (G.R.A.-G.); jacasano@uacam.mx (J.A.C.-S.); 2 Dentistry Department, Campus Campeche, Vizcaya of the Americas University, Campeche 24099, Mexico; 3 Academic Area of Dentistry, Health Sciences Institute, Autonomous University of the State of Hidalgo, Pachuca 42160, Mexico; hislasg@uaeh.edu.mx (H.I.-G.); cdsoniamr@hotmail.com (S.M.-R.); martha_mendoza2138@uaeh.edu.mx (M.M.-R.); cdrubendelarosa@hotmail.com (R.d.l.R.-S.); josedejesusnavarrete@hotmail.com (J.d.J.N.-H.); 4 Universidad Contemporánea de las Américas, Ciudad de Mexico 04890, Mexico; bevada.008@unicla.edu.mx; 5 Center for Advanced Studies and Research on Dentistry Dr. Keisaburo Miyata, School of Dentistry, Autonomous University of the State of Mexico, Toluca 50130, Mexico; 6 Richard M. Fairbanks School of Public Health, Indiana University/Purdue University, Indianapolis, IN 46202, USA; gmaupome@iu.edu; 7 Indiana University Network Science Institute, Bloomington, IN 47408, USA

**Keywords:** oral health, toothbrushing, health inequalities, schoolchildren

## Abstract

Periodic toothbrushing is the most common, effective, and reliable way to mechanically remove biofilm from oral tissues. The objective of the present study was to determine the association between toothbrushing frequency and socioeconomic position for schoolchildren between 6 and 12 years of age in four cities in Mexico. A cross-sectional study was conducted on 500 Mexican schoolchildren between 6 and 12 years of age from public schools in four Mexican cities. Questionnaires were administered to the parents/guardians of the schoolchildren to obtain the variables included in the study. The dependent variable was toothbrushing frequency, dichotomized as: 0 = less than twice a day and 1 = at least twice a day. The analysis was performed in Stata. The average age of the schoolchildren was 8.9 ± 1.9 years; 50.4% were female. The prevalence of toothbrushing was 52.8% (at least twice a day) (95% CI = 48.4−57.1). In the multivariate model, the variables associated (*p* < 0.05) with toothbrushing frequency were older age of the schoolchild (OR = 1.14); younger age of the mother (OR = 0.93); being a girl (OR = 1.70); being enrolled in Seguro Popular (OR = 0.69); being in a household that was owned (OR = 2.43); and being a schoolchild who lived in a home that owned a car (OR = 1.31). The prevalence of toothbrushing at least twice a day was just over 50% in these Mexican children. We found demographic and socioeconomic variables to be associated with toothbrushing. Based on socioeconomic variables that were associated with toothbrushing frequency—such as health insurance, home ownership and the household owning a car—the results of the present study confirm the existence of health inequalities in toothbrushing frequency.

## 1. Introduction

Oral diseases are a worldwide public health problem affecting more than 3.5 billion people and are considered to represent a heavy burden of disease [1,2]. Their consequences on quality of life indicate a strong need for attention from health care systems worldwide [3,4,5]. Caries and periodontal diseases, two of the main oral diseases, affect a large percentage of the population in Latin America and Mexico; substantial oral health care needs have been observed [6,7,8].

Dental biofilm is composed of a wide variety of microorganisms; microbial dysbiosis in this biofilm contributes to the occurrence of both caries and periodontal diseases [9,10]. Based on epidemiological and experimental studies, oral health care providers recommend that effective oral hygiene, through the mechanical removal of the biofilm, be conducted to maintain optimal oral health [11]. Periodic toothbrushing is the most common, effective, and reliable way to mechanically remove biofilm from oral tissues [12,13,14] and is therefore one of the educational messages given to children, adolescents, and adults in oral health promotion programs [15,16]. Brushing of the teeth, tongue and gums should be undertaken twice daily. One of these is required to be done before bedtime, although ideally it should be done within 30 min after each meal [17].

Scientific reports from diverse locations in the world have found an association between socioeconomic position and oral/general health [18]. The burden of oral diseases has a disproportionate impact on the poorest and least educated members of society. As oral diseases are expensive to treat, the economic impact on the most socially disadvantaged sector of the population is more deleterious than that on their more advantaged counterparts [19]. Scientific evidence suggests that there is a very strong and consistent association between socioeconomic position (SEP) and the prevalence and severity of oral diseases, a phenomenon called “social gradient in health” [20]. This observation has been replicated around the world for toothbrushing frequency, because individuals of lower socioeconomic status practice less frequent toothbrushing [21,22,23,24,25,26,27]. In addition to SEP, other variables have been observed to be associated with toothbrushing frequency, such as sociodemographic and behavioral variables [15,24,28,29,30,31].

The importance of identifying the variables associated with toothbrushing lies in the fact that prevention strategies can be designed while focusing accurately on the aspects that are susceptible to modification, how to do it, and in what combination. The objective of the present study was to determine the association between toothbrushing frequency and socioeconomic position in schoolchildren between 6 and 12 years of age in four cities in Mexico. The main hypothesis was that some SEP variables would be associated with higher toothbrushing frequency.

## 2. Materials and Methods

### 2.1. Study Design, Population and Sample

A cross-sectional study was conducted in public elementary schools in four Mexican cities: Pachuca, San Luis Potosi, Tepatitlan, and Toluca. From each of the cities, the percentage of students was selected according to the size of the school population of the city, using random sampling. This report is part of a larger project where various indicators of oral health in schoolchildren, costs of caries care [32], and dental trauma [33] were measured. Data collection took place during 2019. Inclusion criteria were being either male or female, aged 6 to 12 years, enrolled in one of the selected schools, mother/guardian signing informed consent, and with the child’s assent. Those schoolchildren who refused to undergo the clinical examination were excluded.

The parameter of interest for calculating sample size was the prevalence of caries. Schools were selected according to a probability proportional to the number of students. The number of schools per stratum was proportional to the total students in the stratum, having at least two schools per locality. We used the following formula to determine sample size:

Estimate of proportions:

$$n=Z^{2}\frac{p\left(1-p\right)}{d^{2}k}\left(1+p\left(k-1\right)\right)$$

where *p* was the proportion of children with at least one decayed tooth, *d* the half-width of the confidence interval, *p* the intra-conglomerate correlation coefficient, *k* the number of students per school and *Z* = 1.96 the quantile 97.5% of a standard normal distribution. The value of *p* was 60%; we assumed *p* = 0.24 and semi-amplitude (*d*) = 7, and 10% estimated losses, with a final sample of 512 students. Finally, the included participants from each city were proportional to the total number of students in each city. Schoolchildren distribution across cities is in Table 1. Mean participation percentage was 88.9% (ranging from 76.8% in Tepatitlan to 95.8% in Pachuca). The final sample was 500 schoolchildren.

### 2.2. Collection of Variables

The selected children were given an informed consent form for their parents/guardians to sign so that their child could be included in the study. A self-administered survey was also sent to collect sociodemographic and socioeconomic data.

The dependent variable was toothbrushing frequency, which was dichotomized as: 0 = less than twice a day and 1 = at least twice a day. The independent variables included in the study were: age of child (in years); age of mother (in years); age of father (in years); number of children in family; sex of child (0 = male, 1 = female); age when toothbrushing started (0 = before 2 years of age, 1 = after 2 years of age); whether child had a dental visit in the last year (0 = never, 1 = not in the last year, 2 = yes in the last year); having health insurance (0 = with insurance, any type (public or private); 1 = Seguro Popular, which is a public insurance for poor families; 2 = uninsured); status of home ownership (0 = borrowed or rented; 1 = owned, paid or to be paid); and whether a car was owned in the household (0 = no; 1 = yes); SEP; housing characteristics and household goods (in tertiles, from the poorest to the richest).

Two SEP indicators were constructed, one based on the ownership of household goods and the other on housing characteristics. Principal component analysis was used, specifically polychoric correlation analysis [34], which allows the incorporation of interrelated categorical variables for the construction of a single indicator variable. Tertiles were calculated for the variables generated, in which the first tertile referred to the group with the worst socioeconomic status and the third tertile to the group with the best status.

### 2.3. The Mexican Health System

The Mexican Health System is a mixed and fragmented health system. The Mexican health system comprises two sectors, public and private. Within the public sector are social security institutions as well as the safety net institutions and programs that serve the population without health insurance. Social security institutions provide medical services to salaried workers and their families, while safety net institutions provide access to health services to individuals who are not compulsorily affiliated with the social security system. In 2003, the Mexican government implemented Seguro Popular (SP), a public health insurance plan that provided partial coverage to its affiliates, funded by the federal and state governments, as well as by some contributions by insureds. For households in the three lowest income deciles, SP was free. In 2020, SP disappeared and was replaced by the Instituto de Salud para el Bienestar (INSABI), as a result of amendments to the General Health Law. The private sector comprises insurance companies and service providers working in private pharmacies, doctors’ offices, clinics and hospitals, including alternative medicine service providers [35,36,37].

### 2.4. Statistical Analysis

In the univariate analysis, measures of central tendency and dispersion were calculated for quantitative variables. Frequencies and percentages were calculated for qualitative variables. In the bivariate and multivariate analyses, the binary logistic regression model was used. The strength of the association between the dependent variable and the independent variables was expressed as odds ratio (OR) with 95% confidence intervals (95% CI). The variance inflation factor (VIF) test was performed to analyze and, if necessary, to avoid multicollinearity between the independent variables. For the construction of the final multivariate logistic regression model, variables that in the bivariate analysis showed a value of *p* < 0.25 were taken into account. The overall fit of the model was performed using the goodness-of-fit test [38]. In the multivariate logistic regression model, confidence intervals were calculated using Huber–White robust standard errors to obtain valid estimates, considering the correlation within groups (children who share the feature of living in the same city, i.e., cluster of the city variable). The latter procedure was undertaken because a child from a given city could be more similar to another child in the same city, compared to those children from other cities (difference between clusters) [39]. There were some missing data for the independent variables. The missing data were imputed with regression imputation. Statistical analysis was performed using the statistical software Stata 16.

### 2.5. Ethical Considerations

This project was approved by the ethics committee of the Autonomous University of the State of Hidalgo (CEEI 000019-2019). It complies with the research requirements and laws in force in Mexico and was conducted in accordance with the Declaration of Helsinki of 1975, as revised in 2013. The mother or guardian of those individuals in the study signed a written informed consent form.

## 3. Results

The study included 500 schoolchildren aged 6 to 12 years. Table 2 shows the results of the descriptive analysis of the study variables. The average age was 8.9 ± 1.9 and 50.4% were female. A considerable proportion, 42.4% (*n* = 212), of the schoolchildren started brushing their teeth before the age of 2. We found that 8.6% (*n* = 43) had never visited the dentist in their life. The majority (81.6%, *n* = 408) of the schoolchildren had some type of health insurance. Of the mothers, 21.4% (*n* = 107) and of the fathers, 23.0% (*n* = 115) had a level of education higher than high school.

In the variables related to socioeconomic position (SEP), it was observed that the majority of the families (56.8%, *n* = 284) lived in a house of their own (paid or to be paid). Most households (55.0%, *n* = 275) owned a car. Equally, Both the “household characteristics” and “household goods” variables are described in Table 1.

Table 1 shows the distribution of toothbrushing frequency; the prevalence of self-reported toothbrushing “At least twice a day” was 52.8% (*n* = 264) (95% CI = 48.4−57.1).

### 3.1. Bivariate Analysis

Table 2 presents the result of the bivariate analysis; the variables associated (*p* < 0.05) with toothbrushing frequency were: age of the mother (OR = 0.97, 95% CI = 0.95−0.98; *p* = 0.001), sex of the child (OR = 1.61, 95% CI = 1.08−2.41; *p* = 0.018), and age at initiation of toothbrushing (OR = 1.53, 95% CI = 1.30−1.79; *p* < 0.001). In the same Table 3 are the variables related to SEP that were associated (*p* < 0.05) with toothbrushing frequency. In the bivariate logistic regression analysis, home ownership (OR = 1.28, 95% CI = 1.01−1.63; *p* = 0.035), car in the household (OR = 1.51, 95% CI = 1.27−1.80; *p* < 0.001), and SEP of household characteristics (OR = 2.60, 95% CI = 1.08−6.22; *p* = 0.032) were the variables related to toothbrushing frequency.

### 3.2. Multivariate Analysis

Table 4 shows the multivariate logistic regression analysis. It was observed that for each year that the age of the schoolchild increased, the likelihood of brushing teeth more frequently increased by 14% (95% CI = 1−30%, *p* = 0.043). For each year that the mother’s age increased, the likelihood of brushing teeth more frequently decreased by 7.5% (*p* < 0.05). The likelihood of brushing teeth more frequently was 1.70 (95% CI = 1.05−2.75) times higher among girls, than among boys. Those who were enrolled in SP brushed their teeth less frequently (OR = 0.69, 95% CI = 0.50−0.95) than those who had public or private health insurance. Regarding the SEP variables, those with their own home were more likely (OR = 2.43; 95% CI = 1.10−5.33) to brush their teeth more frequently than those who lived in a borrowed/rented home. Finally, children whose home owned a car were more likely to brush their teeth more frequently (OR = 1.31; 95% CI = 1.21−1.43).

## 4. Discussion

The objective of the present study was to determine the association between toothbrushing frequency and socioeconomic position in schoolchildren between 6 and 12 years of age in four cities in Mexico. The prevalence of toothbrushing at least two or more times a day was 52.8%; some demographic and socioeconomic variables were related to toothbrushing frequency. Studies in Mexico have reported prevalence ranging from 49.8% in Campeche [29], 56.3% in Sinaloa [40], to 81.7% also in Campeche [15]. In other countries, very low percentages of toothbrushing in schoolchildren have been reported, such as in Iran (18.0%) [26], Palestine (19.7%) [41], or China (26.3%) [42], to prevalence numbers higher than 80%, such as in Norway [27], Indonesia [43,44], and Brazil [23]. Differences may be due to different circumstances such as the specific age of the children and adolescents in the studies, and even the economic development of the communities or countries where research was conducted.

Within the mosaic of factors that are associated with toothbrushing frequency, one of the variables that plays an important role is socioeconomic position. As discussed here, studies about the frequency of brushing and SEP in school-age children conducted in Mexico and elsewhere also found that SEP variables were associated with higher frequency of brushing, for example, that the child was enrolled in a private school, had social security or private insurance, as well as higher socioeconomic level as measured by level of education, occupation of both parents and owning a car in the family [40], the schooling level of the mother [15,45], or the size of the family [29]. We confirmed features identified in previous literature reports; for example, a study in Norway [27] found that girls and boys whose parents had a higher educational level had favorable oral health behaviors; they brushed their teeth more frequently than other children. In Portugal [24], adolescents living in rural areas and with lower educational level brushed their teeth less than twice a day. Moreover, lower educational level of the father and less favorable employment status of the mother were also associated with lower frequency of toothbrushing. For adolescents in Iran [26], higher educational level of the mother and better job status of the father were SEP variables associated with greater toothbrushing frequency. Using data from several national surveys in Korea, Kim and Kang [24] conducted an analysis in adolescents in which they compared the average frequency of toothbrushing per day. The average frequency of toothbrushing per day was lower in those with low educational level, the unemployed, and those living in rural areas. Another study in adolescents [22] in Slovakia showed an association between the absence of daily toothbrushing and lower socioeconomic status. In Australia [21], children who had private health insurance exhibited higher frequency of toothbrushing. Paradoxically, in Brazil [23] low frequency of daily toothbrushing was associated with higher level of education of the mother. There is no “ideal indicator” related to SEP for all purposes and applicable to all life stages and in all settings. Each indicator measures different aspects and may be more or less relevant to different health responses throughout an individual’s life. While a single measure of SEP may demonstrate an association with a health response, it will not encompass the entirety of the effect of SEP on health. This is why it is common in epidemiological research to use a variety of SEP indicator variables [46,47]. All in all, low SEP during childhood tends to be associated with lower toothbrushing frequency and higher caries prevalence among adults. Positive SEP changes in adulthood are not sufficient to offset earlier adverse effects [48].

Our findings showed that girls are more likely to brush their teeth more frequently than boys, as found in Brazil [24] and Greece [30]. These findings could be explained by the subjective perception that females tend to be more concerned about their body and appearance; therefore, they might be more willing to adopt behaviors and habits that promote their oral health [30,49].

Toothbrushing appears to be a very complex behavior determined by multiple factors. The age at which children can handle their own toothbrush has been determined based on different factors, such as the age at which the child shows interest in brushing, the maturation of neuromuscular function, and developmental stage of sensory functions [50]. Regarding the age of the individuals included in the present study, other studies that considered the same age group also found that toothbrushing frequency was higher in older children [15,28,29]. This may be due to the psychomotor development of the individual, although it is difficult to define precisely the age at which children can manage their own toothbrushing. It has been suggested that the age at which parents should let their children brush their teeth on their own is 7 to 8 years old [50]. Other authors have indicated that toothbrushing programs should take into account not only the needs presented but also the age of the target individuals [51]. In the present study, we found that as mother’s age increased, toothbrushing frequency for the child decreased, regardless of other factors. The mother’s characteristics have an influence on various health events of her children. This has been observed by other authors [15,31]. Young children depend on their parents for their health behaviors, daily hygiene, lifestyle habits, and oral health maintenance, including toothbrushing, eating habits, and dental visits. Caries prevention is derived from a favorable balance between the investment of resources, particularly money and time, and benefits obtained [31].

Among the contributions of the present study is the use of a representative random sample of schoolchildren from different cities in Mexico, which gives greater generalizability to the results compared to studies that recruit children from dental clinics or schools. However, the study has limitations that must be taken into account in order to adequately interpret the results. First, there is its design: like all cross-sectional studies, it poses the problem of temporal ambiguity, which refers to measuring cause and effect at the same time. For this reason, causal relationships cannot be established, but rather statistical associations. Moreover, although self-reporting of health events has been commonly used in epidemiological studies, this approach to data collection may introduce some bias if participants prefer to report behaviors that they perceive as socially correct, or if they overestimate certain events in memory. Finally, this is not a national study but an in-depth assessment of four mid-size cities.

Future directions derived from our research should formally examine the effectiveness of health promotion campaigns and social marketing to improve tooth brushing frequency, to ascertain impact with and without public health support to supply toothpaste and toothbrushes across SEP groups.

## 5. Conclusions

The prevalence of toothbrushing (at least twice a day) in these Mexican children was just over 50%. We found that demographic and socioeconomic variables were associated with toothbrushing. Based on socioeconomic variables that were associated with toothbrushing frequency—such as health insurance, home ownership and household owning a car—the results of the present study confirm the existence of health inequalities in toothbrushing frequency.

## Figures and Tables

**Table 1:** Schoolchildren distribution across four cities included in the study.

City	n	%
Tepatitlan	43	8.6
Pachuca	107	21.4
Toluca	112	22.4
San Luis Potosí	238	47.6
	500	100

**Table 2:** Descriptive analysis of the variables included.

	Mean ± SD	Minimum-Maximum
Age of child	8.92 ± 1.99	6−12
Mother’s age	34.55 ± 6.08	21−54
Father’s age	36.98 ± 6.63	22−57
Number of children	3.27 ± 0.98	1−6
	Frequency	Percentage
Sex		
Boys	248	49.6
Girls	252	50.4
Age at initiation of toothbrushing		
Before 2 years of age	212	42.4
After 2 years of age	288	57.6
Dental visit		
Never in lifetime	43	8.6
Not in the last year	270	54.0
Yes, in the last year	187	37.4
Health insurance		
With insurance	408	81.6
Seguro Popular	41	8.2
Uninsured	51	10.2
Home ownership		
Borrowed or rented	216	43.2
Owned (paid or to be paid)	284	56.8
Car in the household		
No	225	45.0
Yes	275	55.0
Household characteristics		
1st tertile (poorest)	192	38.4
2nd tertile	148	29.6
3rd tertile (richest)	160	32.0
Household goods		
1st tertile (poorest)	167	33.4
2nd tertile	186	37.2
3rd tertile (richest)	147	29.4
Toothbrushing frequency		
Less than twice a day	236	47.2
At least twice a day	264	52.8

**Table 3:** Bivariate analysis between toothbrushing frequency and the sociodemographic and oral health variables included in the study.

	OR 95% CI	*p*-Value
Age of child	1.04 (0.98−1.11)	0.169
Mother’s age	0.97 (0.95−0.98)	0.001
Father’s age	0.97 (0.94−1.00)	0.078
Number of children	0.94 (0.77−1.14)	0.538
Sex		
Boys	1 *	
Girls	1.61 (1.08−2.41)	0.018
Age at initiation of toothbrushing		
Before 2 years of age	1 *	
After 2 years of age	1.53 (1.30−1.79)	0.000
Dental visit		
Never in lifetime	1 *	
Not in the last year	1.97 (0.86−4.50)	0.104
Yes, in the last year	0.68 (0.28−1.63)	0.394
Health insurance		
With insurance	1 *	
Seguro Popular	0.72 (0.46−1.11)	0.145
Uninsured	0.68 (0.24−1.91)	0.473
Mother’s educational level		
Up to junior high school	1 *	
Up to high school	1.28 (0.51−3.21)	0.586
Higher than high school	0.55 (0.23−1.27)	0.166
Father’s educational level		
Up to junior high school	1 *	
Up to high school	1.54 (0.81−2.92)	0.185
Higher than high school	0.73 (0.30−1.80)	0.501
Home ownership		
Borrowed or rented	1 *	
Owned (paid or to be paid)	1.28 (1.01−1.63)	0.035
Car in the household		
No	1 *	
Yes	1.51 (1.27−1.80)	0.000
Household characteristics		
1st tertile (poorest)	1 *	
2nd tertile	1.64 (0.82−3.30)	0.160
3rd tertile (richest)	2.60 (1.08−6.22)	0.032
Household goods		
1st tertile (poorest)	1 *	
2nd tertile	1.30 (0.79−2.15)	0.295
3rd tertile (richest)	4.03 (0.57−28.03)	0.159

* Reference category.

**Table 4:** Multivariate analysis between toothbrushing frequency and the independent variables included in the study.

	OR 95% CI	*p*-Value
Age of child	1.14 (1.01−1.30)	0.043
Mother’s age	0.93 (0.90−0.97)	0.003
Sex		
Boys	1 *	
Girls	1.70 (1.05−2.75)	0.030
Health Insurance		
With insurance	1 *	
Seguro Popular	0.69 (0.50−0.95)	0.026
Uninsured	0.95 (0.51−1.76)	0.887
Home ownership		
Borrowed or rented	1 *	
Owned (paid or to be paid)	2.43 (1.10−5.33)	0.026
Car owned by the household		
No	1 *	
Yes	1.31 (1.21−1.43)	0.000

* Reference category. Goodness of fit test: Hosmer–Lemeshow chi2 (8) = 7.10; *p* = 0.5263.

## Data Availability

The datasets generated during and/or analyzed during the current study are available from the corresponding author on reasonable request.
